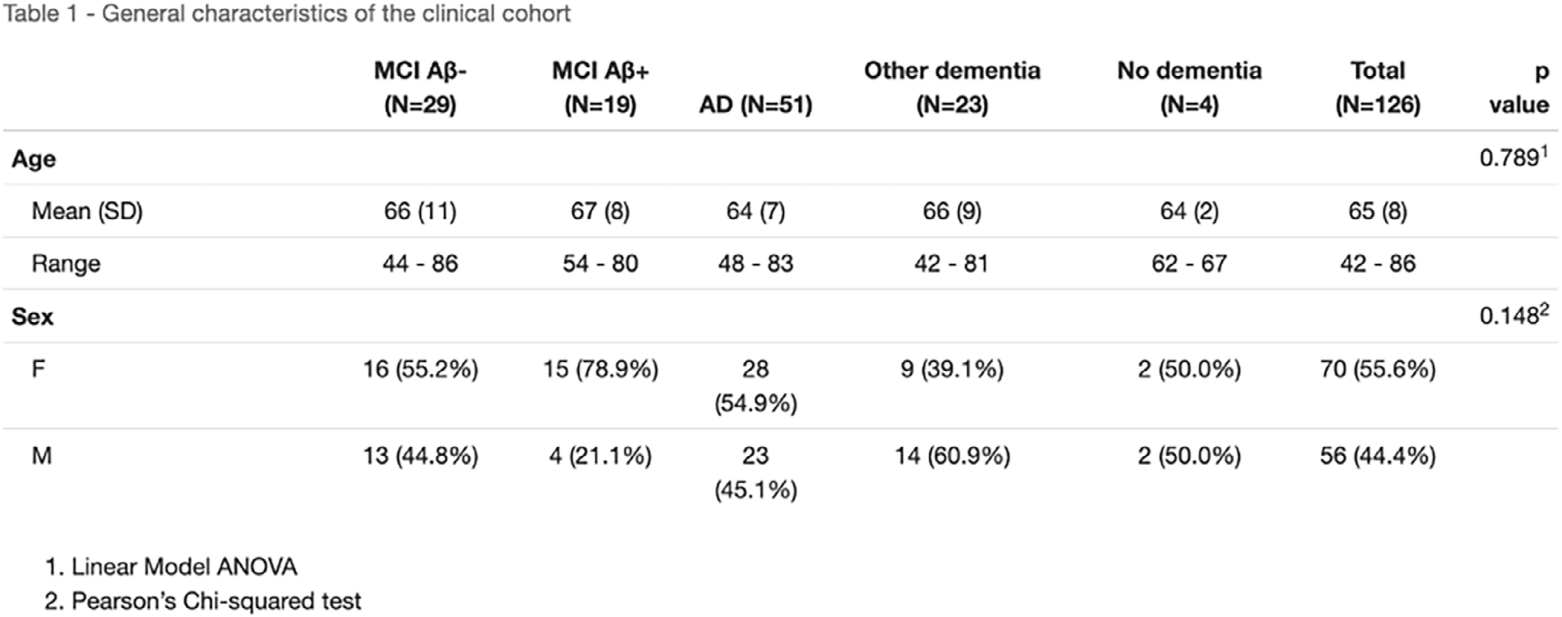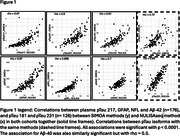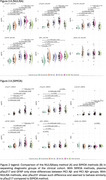# Comparison of NULISAseq and SIMOA methods in plasma biomarker analysis in two memory clinic and research cohorts

**DOI:** 10.1002/alz70856_107092

**Published:** 2026-01-08

**Authors:** Marco Bucci, Marina Bluma, Mariola Zapater‐Fajari, Irina Savitcheva, Konstantinos Chiotis, Nenad Bogdanovic, Guglielmo Di Molfetta, Ilaria Pola, Kübra Tan, Wiebke Traichel, Andrea Benedet, Nicholas Ashton, Kaj Blennow, Henrik Zetterberg, Agneta K Nordberg

**Affiliations:** ^1^ Department of Neurobiology, Care Sciences and Society, Division of Clinical Geriatrics, Center for Alzheimer Research, Karolinska Institutet, Stockholm, Sweden; ^2^ Turku PET Centre, Turku University Hospital, University of Turku and Åbo Akademi University, Turku, Finland; ^3^ Department of Neurobiology, Care Sciences and Society, Division of Clinical Geriatrics, Center for Alzheimer Research, Karolinska University, Stockholm, Sweden; ^4^ Medical Radiation Physics and Nuclear Medicine, Karolinska University, Stockholm, Sweden; ^5^ Department of Neurology, Karolinska University Hospital, Stockholm, Sweden; ^6^ Theme Inflammation and Aging, Karolinska University Hospital, Stockholm, Sweden; ^7^ Department of Psychiatry and Neurochemistry, Institute of Neuroscience and Physiology, The Sahlgrenska Academy, University of Gothenburg, Mölndal, Sweden; ^8^ Clinical Neurochemistry Laboratory, Sahlgrenska University Hospital, Mölndal, Västra Götaland län, Sweden; ^9^ Wisconsin Alzheimer's Disease Research Center, University of Wisconsin‐Madison, School of Medicine and Public Health, Madison, WI, USA; ^10^ Department of Neurodegenerative Disease, UCL Institute of Neurology, Queen Square, London, United Kingdom; ^11^ UK Dementia Research Institute, University College London, London, United Kingdom; ^12^ Hong Kong Center for Neurodegenerative Diseases, Hong Kong, Hong Kong, China

## Abstract

**Background:**

The diagnostic work‐up of Alzheimer's disease (AD) requires reliable and accurate diagnostic biomarkers to aid clinicians in making the correct diagnosis. Plasma biomarker analysis methods are emerging as cost‐effective and possibly reliable tools. The NULISAseq (Alamarbio) CNS panel has recently been commercialized and has not yet been extensively tested with previously used assays. We aimed to compare a selection of biomarkers assayed with NULISAseq against more established SIMOA methods in a memory clinic and a research cohort from Karolinska.

**Method:**

A total of 176 subjects from a memory clinic cohort and a research cohort were included in this study. The clinical cohort from the Karolinska Hospital Memory Clinic included 126 patients (70W/56M, mean age 65±8 years, range 42‐86), who, after extensive investigations (including Amyloid‐PET imaging), were assigned to the following diagnostic groups: MCI Aβ+(*n* = 19), MCI Aβ‐(*n* = 29), AD(*n* = 51), other dementia(*n* = 23) and no dementia(CU)(*n* = 4). Further details on the general characteristics are presented in Table 1. The research cohort (ROADAD) included 30 men and 20 women (mean age 64±13 years, range 28‐86). For all 176 cases, plasma samples, taken during the investigations, were processed with NULISAseq to obtain pTau217, pTau231, pTau181, GFAP, NFL, Aβ42 and Aβ40 levels. Additionally, SIMOA assays (Quanterix‐N4PE and AlzPath) were also applied. SIMOA measurements for pTau231 and pTau181 were not available for the ROADAD cohort.

**Result:**

In the entire sample, the two methods generally showed strong agreement (Figure 1) (Spearman's rho>=0.8 for pTau isoforms and GFAP). pTau217 vs pTau231 had rho=0.7 for SIMOA, while rho=0.86 for NULISA. When the two panels of measurements were compared in the clinical cohort to evaluate differences between diagnoses(Figure 2), pTau217 and GFAP levels were significantly different between MCI Aβ‐ and MCI Aβ+ regardless of the method. Comparing the diagnostic groups, pTau231 was more similar to pTau217 with the NULISA than with SIMOA method. pTau181 showed more pronounced differences within the AD spectrum with NULISA than SIMOA.

**Conclusion:**

Both methods show similar results and trends, but NULISAseq produced more distinct group differences. It remains to be understood if the closer relation between pTau217 and pTau231 with NULISA is a benefit or a disadvantage compared with SIMOA methods.